# Heat-induced proteomic changes in anthers of contrasting rice genotypes under variable stress regimes

**DOI:** 10.3389/fpls.2022.1083971

**Published:** 2023-01-13

**Authors:** Ritesh Kumar, Arindam Ghatak, Isha Goyal, Neelam K. Sarkar, Wolfram Weckwerth, Anil Grover, Palak Chaturvedi

**Affiliations:** ^1^ Department of Plant Molecular Biology, University of Delhi, New Delhi, India; ^2^ Molecular Systems Biology Lab (MOSYS), Department of Functional and Evolutionary Ecology, University of Vienna, Vienna, Austria; ^3^ Vienna Metabolomics Center (VIME), University of Vienna, Vienna, Austria

**Keywords:** anthers, heat stress, LC-MS/MS, proteomics, reproductive thermotolerance, rice

## Abstract

Heat stress drastically affects anther tissues resulting in poor plant fertility, necessitating an urgent need to determine the key proteome regulation associated with mature anther in response to heat stress. We identified several genotype - specific protein alterations in rice anthers of Moroberekan (Japonica, heat sensitive), IR64 (Indica, moderately heat tolerant), and Nagina22 (Aus, heat tolerant) in the short-term (ST_HS; one cycle of 42°C, 4 hours before anthesis) and long-term (LT_HS; 6 cycles of 38°C, 6 hours before anthesis) heat stress. The proteins upregulated in long-term heat stress in Nagina22 were enriched in biological processes related to unfolded protein binding and carboxylic acid metabolism, including amino acid metabolism. In short-term heat stress, Nagina22 anthers were enriched in proteins associated with vitamin E biosynthesis and GTPase activator activity. In contrast, downregulated proteins were related to ribosomal proteins. The expression of different Hsp20 and DnaJ was genotype specific. Overall, the heat response in Nagina22 was associated with its capacity for adequate metabolic control and cellular homeostasis, which may be critical for its higher reproductive thermotolerance. This study improves our understanding of thermotolerance mechanisms in rice anthers during anthesis and lays a foundation for breeding thermotolerant varieties *via* molecular breeding.

## 1 Introduction

Heat stress (HS) is one of the major environmental factors influencing the geographical distribution of plants. According to climate change projections, extreme temperatures may negatively affect plant cultivation in the future (http://climate.nasa.gov/evidence/; Chaturvedi et al., 2021). Rice is the most consumed grain in the world. Every degree Celsius increase in global mean temperature reduces rice yields by 3.2% (Zhao et al., 2017). Several physiological processes in rice plants are affected by HS, including stomatal opening, photosynthetic activity, and growth (Jagadish et al., 2014; Kumar et al., 2022). The reproductive stage of the plants is overly sensitive to HS (Zinn et al., 2010; Bokszczanin, 2013; Chaturvedi et al., 2016; Pazhamala et al., 2020; Chaturvedi et al., 2021). The effect of HS on the physiology and genetics of rice plants at the reproductive stage has been studied extensively (Jagadish and Pal, 2009; Tenorio et al., 2013; Jagadish et al., 2014; Das et al., 2015). Rice male reproductive organs like anther and pollen are more sensitive to HS than the female reproductive organs (Imin et al., 2001; Fujita et al., 2006; Prasad et al., 2006; Jagadish et al., 2010; Jagadish et al., 2014; Shi et al., 2022). Recent work has suggested that the female gametophyte is also affected irreversibly by heat stress (Shi et al., 2022). HS reduces fertility and the number of florets per plant in rice (Kobata et al., 2013). The duration of the HS susceptible period during anthesis differs between indica and japonica rice varieties (Satake and Yoshida, 1978; Matsui et al., 2000). Generally, tolerant varieties show early morning anthesis to avoid HS. HS produces poor anther dehiscence, reducing rice pollination (Satake and Yoshida, 1978; Matsui et al., 1997) by inhibiting pollen swelling and the normal function of the thecae (Matsui et al., 2000).

Nagina22 (N22) is a highly heat-tolerant rice variety; thus, it is a good source for mining genes for tolerance against heat and drought stress (Bahuguna et al., 2014; Ye et al., 2012). Early anther dehiscence, while anthers are still inside the glumes, leads to HS avoidance and greater pollination in N22 (Satake and Yoshida, 1978; Mackill et al., 1982). The metabolome and transcriptome analyses have revealed sugar starvation as a major process involved in reproductive failure: higher expression of sugar transporter (MST8) and a cell wall invertase (INV4) were identified in N22 in response to combined heat and drought stress (Li et al., 2015). Jagadish et al. (2010) showed that Moroberekan (Japonica), Indica Rice 64 (IR64, Indica) and N22 (Aus) demonstrated 18%, 48% and 71% spikelet fertility when exposed to 6 h of heat stress (38°C), at anthesis stage, respectively. Two-dimensional gel electrophoresis-based proteome analysis of anthers revealed the presence of 46 proteins, including significant upregulation of the cold-shock and heat-shock proteins in N22 (Jagadish et al., 2010). However, the latter study did not divulge the global perspective of the anther proteomes in these varieties in response to HS. In another study, anther proteome of thermotolerant Dianxi 4 rice genotype (Japonica) stressed at 38°C demonstrated the proteins responsible for maintaining proteostasis, like sHsps, Hsp70 and trehalose synthase, were upregulated (Kim et al., 2015). Interestingly, the comparative proteome between heat-sensitive and heat-tolerant rice varieties are not yet explored.

In the present study, we have used a shotgun proteomics approach to analyze the rice anther proteome in contrasting genotypes such as heat-sensitive Moroberekan, moderately heat-tolerant IR64 and heat-tolerant N22. Our analysis focused on anther dehiscence stage, as this stage is crucial for the fertilization process in rice. The anther proteome changes were identified in response to two stress regimes: short-term heat stress (ST_HS) and long-term heat stress (LT_HS). This study aimed to determine the key regulators involved in heat stress responses to develop rice varieties with better thermotolerance through modern molecular breeding strategies.

## 2 Material and methods

### 2.1 Plant material and growth conditions

**Figure 1:**
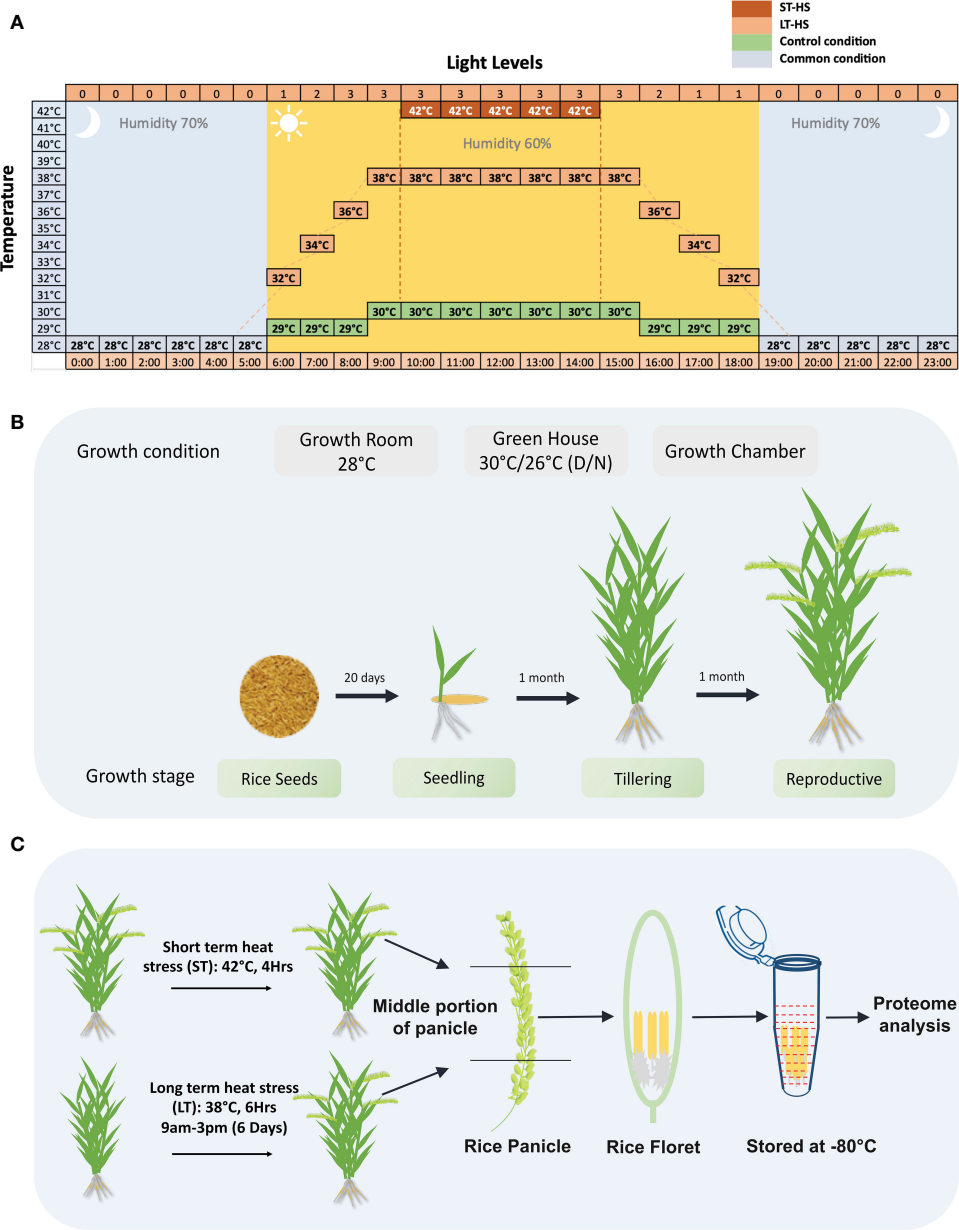
Details of the growth and stress conditions followed in the experiment. **(A)** Illustration showing the stress regimes used in the current study. In the short-term heat stress, plants were exposed to 42°C for 4 hours, and in long-term heat stress, plants were exposed to 38°C temperature for 6 hours. **(B)** Growth conditions for growing rice plants for the study. **(C)** The stress regimes used in ST-HS (short-term heat stress) and LT-HS (long-term heat stress) treatments followed for the proteome analysis are shown. The plants at the reproductive stage were stressed, and the anthers were dissected just before the anthesis.

Three rice genotypes Moroberekan, IR64 and N22 were used in this study. The details of the growth conditions followed in the analysis of Moroberekan, IR64 and N22 rice genotypes, as well as the relationship of the pollen grain stages on the progression to anthesis in rice to the stress regimes followed in this study, are shown in **Figure 1**. Several independent sets of plants were transferred to growth chambers for the HS treatment to obtain plants with a variable time of flowering. For the ST_HS, anthers were harvested at ‘just before anthesis’ stage from plants subjected to one cycle of 42°C (4 h) HS (**Figure 1A**). For the LT_HS, anthers were harvested at a similar growth stage from plants subjected to 6 days of consecutive 38°C (6 h) HS (**Figure 1A**). In LT_HS treatment, the temperature regime followed was 28°C at 6 AM, gradually increased to 38°C by 9 AM, maintained at 38°C till 3 PM and gradually lowered to 28°C and maintained overnight. In the unstressed control (CON) condition, the temperature followed was: 28°C at 6 AM, gradually increased to 30°C and maintained till 6 PM and gradually brought down to 28°C overnight. Plants (15 to 35) were harvested (for each treatment), and flowering spikelets were collected on ice. The spikelets were dissected to separate the pollinated pistils and anthers. The anthers were harvested from only the middle portion of the whole spikelet to maintain uniformity in the development stage. Dissected anthers were collected in tubes containing liquid nitrogen and stored at –80°C until further use (**Figures 1B, C**).

### 2.2 Protein isolation and western blotting analysis

Harvested anthers (~20 mg) were lysed and homogenized in TRIzol™ Reagent (Invitrogen™) to extract proteins for Western blotting. Extracted proteins were dissolved in 0.25% SDS and protein was quantified using Bradford reagent. For Western blotting, 10 μg of total soluble protein was loaded on the SDS-gel. Proteins were transferred to the nitrocellulose membrane (Amersham, UK). Hsp101 protein was probed using polyclonal anti-AtHsp101/ClpB primary antibodies (#AS08283, Agrisera, Sweden) and horseradish peroxidase (HRP) -conjugated anti-rabbit secondary antibodies (Sigma-Aldrich, USA), both at 1:10000 dilutions. The same membrane was probed with anti-tubulin alpha chain antibodies (# AS10680, Agrisera) for loading control. The blots were developed by enhanced chemiluminescence (ECL).

### 2.3 Protein extraction and pre-fractionation

Proteins were extracted and quantified using a shotgun proteomics approach (Chaturvedi et al., 2013; Valledor and Weckwerth, 2014; Chaturvedi et al., 2015). Anther samples were freeze-dried and ground for 2 min in a shaking mill using steel balls (2 mm diameter). The homogenized samples were resuspended in 200 μL of protein extraction buffer (100 mM Tris- HCl, pH 8.0; 5% SDS, 10% glycerol; 10 mM DTT; 1% plant protease inhibitor cocktail (Sigma P9599) and incubated at room temperature for 5 min followed by incubation for 2.5 min at 95°C and centrifugation at 21000 × g for 5 min at room temperature. The supernatant was carefully transferred to a fresh tube. Two hundred microliter of 1.4 M sucrose was added to the supernatant. Proteins were extracted twice with 200 μL TE buffer-equilibrated phenol followed by counter extraction with 400 μL of 0.7 M sucrose. Phenol phases were combined and mixed with 2.5 volumes of 0.1 M ammonium acetate in methanol to precipitate proteins. After 16 h of incubation at -20°C, samples were centrifuged for 5 min at 5000 × g. The pellet was washed twice with 0.1 M ammonium acetate, once with acetone and air-dried at room temperature. The pellet was re-dissolved in 6 M urea and 5% SDS and protein concentration were determined using the bicinchoninic acid assay. Proteins were pre-fractionated by SDS-polyacrylamide gel electrophoresis. Forty micrograms of total protein were loaded onto a gel and run for 1.5 cm. Gels were fixed and stained with methanol: acetic acid: water: Coomassie Brilliant Blue R-250 (40:10:50:0.001). Gels were destained in methanol: water (40:60), and then each lane was divided into two fractions (Ghatak et al., 2021).

### 2.4 Protein digestion and LC-MS/MS

Gel pieces were destained, equilibrated and digested with trypsin (using Trypsin Sequencing Grade from Roche [11418475001]), desalted employing Bond-Elute C-18 SPEC plate (Agilent Technologies, Santa Clara, CA, USA) and concentrated in a Speed Vac concentrator (SCANVAC Cool Safe 110-4, Speed Vacuum concentrator, Labogene). Prior to mass spectrometric measurement, the tryptic peptide pellets were dissolved in 4% (v/v) acetonitrile, 0.1% (v/v) formic acid. One µg of the digested peptide (2 biological replicates from each condition) was loaded on a C18 reverse-phase column (Thermo Scientific, EASY‐Spray 500 mm, 2 µm particle size). The separation was achieved with a 90 min gradient from 98% solution A (0.1% formic acid) and 2% solution B (90% ACN and 0.1% formic acid) at 0 min to 40% solution B (90% ACN and 0.1% formic acid) at 90 min with a flow rate of 300 nl min^−1^. nESI‐MS/MS measurements were performed on Orbitrap Elite (Thermo Fisher Scientific, Bremen, Germany) with the following settings: Full scan range 350–1800 m/z resolution 120000 max. 20 MS2 scans (activation type CID), repeat count 1, repeat duration 30 s, exclusion list size 500, exclusion duration 30 s, charge state screening enabled with the rejection of unassigned and +1 charge states, minimum signal threshold 500.

### 2.5 Peptide and protein identification

For protein identification, raw data were searched with the SEQUEST algorithm present in Proteome Discoverer version 1.3 (Thermo, Germany) as described in (Valledor and Weckwerth, 2014) using protein FASTA. We have used the following settings in Proteome Discoverer for data analysis which include: Peptide confidence: High, which is equivalent to 1% FDR, and Xcorr of 2, 3, 4, 5, 6 for peptides of charge 2, 3, 4, 5, 6. The variable modifications were set to acetylation of the N-terminus and oxidation of methionine, with a mass tolerance of 10 ppm for the parent ion and 0.8 Da for the fragment ion. The number of missed and/or non-specific cleavages permitted was 2. There were no fixed modifications, as dynamic modifications were used. The identified proteins were quantitated based on total ion count, followed by an NSAF normalization strategy (Paoletti et al., 2006).


(NASF)κ=(PSM/L)κ/∑i=1N(PSM/L)i

The total number of spectra counts for the matching peptides from protein k (PSM) was divided by the protein length (L), then divided by the sum of PSM/L for all N proteins.

All the MS/MS spectra of the identified proteins and their meta-information were further uploaded to the PRIDE repository, Project accession: PXD035952.

### 2.6 Statistics for proteome data analysis and bioinformatics

A t-test was applied to find significantly altered proteins between control and stress condition in each genotype, and proteins with *p*-value < 0.05 were further considered. Differentially expressed proteins (DEPs), fold change (FC) were calculated for all identified proteins in HS compared to CON. Proteins with FC ≥ 2 (upregulation) or ≤ 0.5 (downregulation) were considered. Gene ontology was determined using RiceNetDB (http://bis.zju.edu.cn/ricenetdb/documentation.php). Only gene ontology (GO) terms with a *p*-value ≤ 0.01 were used for the analysis. Gene ontology network analysis for the identified DEPs was performed using Cytoscape software (Version 3.7.1) with ClueGO (Version 2.5.4) plug-in. ClueGo was used to visualize non-redundant biological terms for large clusters of genes in a functionally grouped network. The *Oryza sativa* (Japonica) marker list was used for searching biological processes, molecular function and cellular components related to proteins identified. *P*-value was calculated using a two-sided hypergeometric test and Benjamini-Hochberg adjustment for multiple test corrections. GO terms with P-value<0.05 were considered significant. The protein-protein interactions network in each set of up and down-regulated proteins was generated using the STRING database (Version 11.0). We restricted the parameter with the highest confidence to 0.9 and neglected the single molecules found in the interaction map to best predict the interactions.

## 3 Results

### 3.1 Proteome alteration in anther tissue of the three rice genotypes in response to heat stress

Heat stress demonstrated phenotypic changes in N22 plants where mature spikelets were deformed, and pollen viability was reduced, especially after long-term heat stress compared to short-term heat stress (**Figure S1**).

The detailed lists of the identified proteins in Moroberekan, IR64 and N22, are provided in supporting information (**Tables S1–S3**). In total, proteins identified in Moroberekan were 2689 in control (referred to as MCT for Moroberekan control), 2529 in ST_HS (MST for Moroberekan ST_HS) and 2687 in LT_HS (MLT for Moroberekan LT_HS). Similarly, proteins identified in IR64 were 2739 in ICT (IR64 control), 2690 in IST (IR64 ST_HS) and 2973 in ILT (IR64 LT_HS) and in N22, 3072 proteins were identified in NCT (N22 control), 3009 in NST (N22 ST_HS) and 3045 in NLT (N22 LT_HS) as shown in **Figure S2A**. Thus, the number of identified proteins was in the order of N22>IR64>Moroberekan. The number of unique and common proteins in each rice genotypes is represented using the Venn diagram in **Figure S2B**. Between the control and ST_HS regimes, the number of overlapping proteins was higher in N22 compared to Moroberekan and IR64. More proteins were common in the control and LT_HS in Moroberekan than in the other two rice genotypes. Between ST_HS and LT_HS, overlapping protein numbers were higher in Moroberekan than in the other two rice genotypes. The identified proteome from three rice genotypes was compared with transcripts expressed during different developmental stages of rice plants. For this, co-expression analysis tools were used from the rice genome annotation project database. As an output, a z-score trend plot of the locus IDs of proteins was used. In all the rice genotypes, there was a clear dense peak just above the inflorescence P6 samples (mature pollen stage) in the plot generated using the inflorescence and seed developmental series (GSE6893) dataset and panicle 5 (heading panicle stage) and stamen (stamen 1 day before flowering) in the plot generated using tissue atlas from Minghui 63 rice (GSE19024) (**Figure S3**). These results suggested that the proteins identified in the present work corresponded greatly with the transcripts expressed in anthers of the mature pollen stage.

### 3.2 Identification of differentially expressed proteins (DEPs)

**Figure 2:**
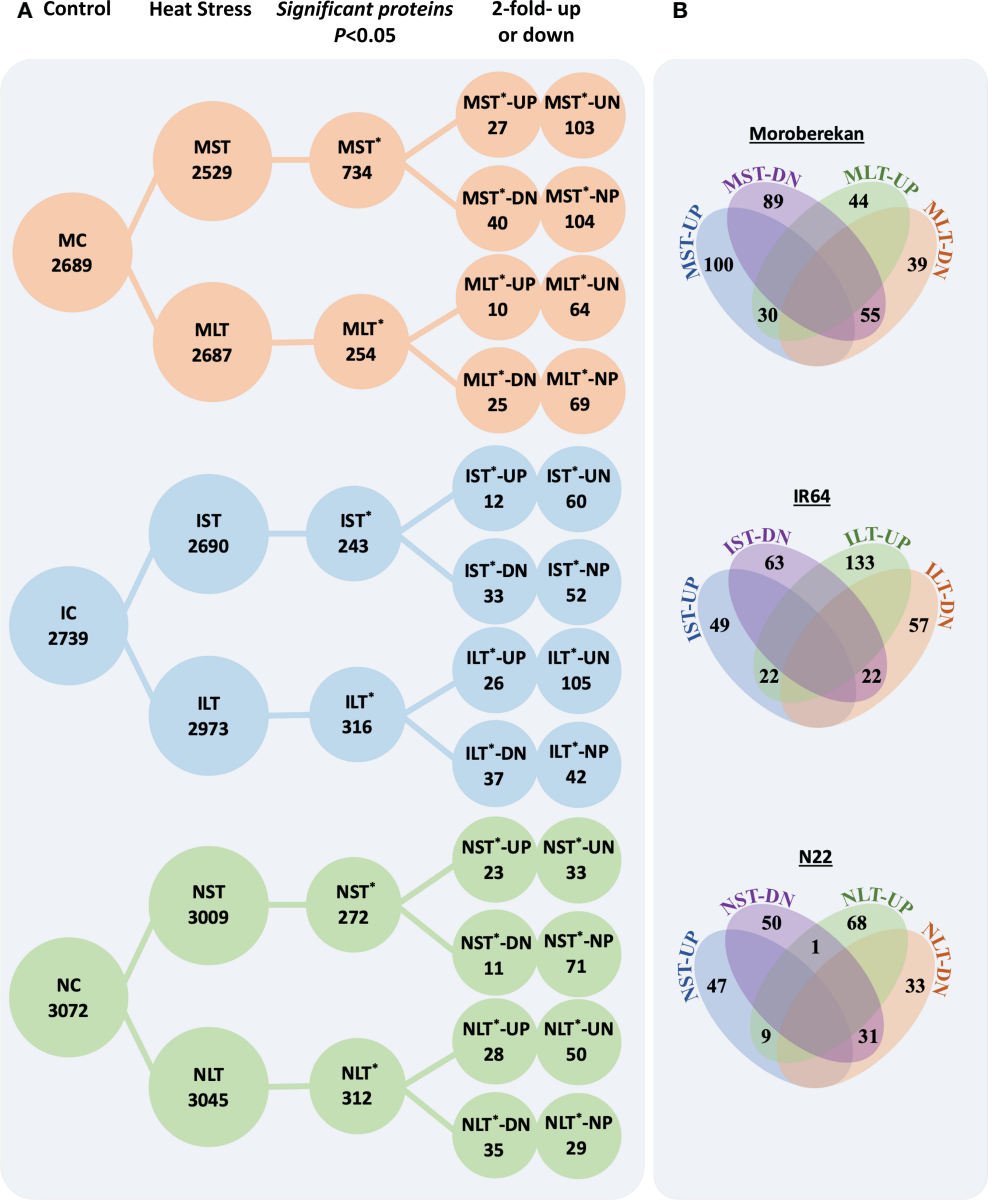
**(A)** The differentially expressed proteins in different rice genotypes under different heat stress regimes. **(B)** Venn diagrams show the overlapping significant and differentially expressed proteins between different datasets in each genotype. ST, Short term heat stress; LT, Long-term heat stress; MC, Moroberekan control; MST, Moroberekan ST_HS; MLT, Moroberekan LT_HS; IC, IR64 control; IST, IR64 ST_HS; ILT, IR64 LT_HS, NC, N22 control; NST, N22 ST_HS; NLT, N22 LT_HS; UP, upregulated; DN, downregulated, *-differentially expressed proteins numbers; UN-uniquely present in HS, proteins present in the heat stress sample but not in control; NP-not present in HS, proteins present in control but not detected in the heat stress condition.

The lists of the differentially expressed proteins (DEPs) in Moroberekan, IR64 and N22 rice types are provided in supporting information **Table S4**. The total identified proteins were sorted into DEPs between the control and stressed samples by applying *p*-value<0.05 (**Figure 2A**). In Moroberekan, 734 DEPs were identified in MST and 254 in MLT. Of the 734 MST DEPs, 130 proteins were upregulated >2 fold, and of these, 103 proteins were exclusively identified in ST_HS. In the same dataset, 144 proteins were downregulated, and of these, 104 proteins were present only in the control but not in the HS condition. Of the 254 MLT DEPs, 74 proteins were upregulated, of which 64 were uniquely identified in LT_HS. In this analysis, 94 proteins were downregulated and of which 69 proteins were exclusively present in the control condition. In IR64, 243 DEPs were identified in IST, out of which 72 were upregulated, and 60 were noted exclusively in IST condition. In the same dataset, 85 DEPs were downregulated, of which 52 were exclusively present only in the control condition. Similarly, in ILT, 316 DEPs were identified, which include 131 upregulated, of which 105 were exclusively present in the LT_HS condition. In the same dataset, 79 DEPs were downregulated, and 42 were exclusively present in the control condition. In N22, 243 DEPs were identified in NST. At FC >2, 56 DEPs were upregulated and 33 were exclusively present in the NST. Under the NST regime, 82 DEPs were downregulated, and 71 were exclusively present in the control condition. Out of 312 DEPs identified in NLT, 78 were upregulated, of which 50 were unique under LT_HS. In the same dataset, 64 DEPs were downregulated, of which 29 were present in control but not in HS condition.

The analysis of the overlapping DEPs among ST_HS and LT_HS datasets showed that in Moroberekan, 30 upregulated proteins were common between MST and MLT treatments. In comparison, 55 downregulated proteins were common between MST and MLT datasets (**Figure 2B**). In IST and ILT, 22 DEPs were common. In NST and NLT datasets, 9 DEPs were commonly upregulated and 31 were downregulated (**Figure 2B**). The number of common DEPs between all three rice genotypes was smaller in ST_HS than in LT_HS treatments. Among the three rice genotypes, there were no common upregulated DEPs, while two common DEPs down-regulated were β-amylase (Os07g35880) and cytochrome P450 (Os02g38290) in the ST_HS treatment. In ST_HS, the numbers of uniquely upregulated DEPs were 127, 66 and 31 in Moroberekan, IR64 and N22, respectively (**Figure S4**). In ST_HS, unique downregulated proteins were 129, 68 and 74 in Moroberekan, IR64 and N22, respectively. Overall, in LT_HS, unique upregulated proteins were 68, 126 and 70 in Moroberekan, IR64 and N22, respectively, and downregulated proteins were 88, 71 and 61 in Moroberekan, IR64 and N22, respectively (**Figure S4**).

### 3.3 Expression pattern of Hsp101 in rice anthers

**Figure 3:**
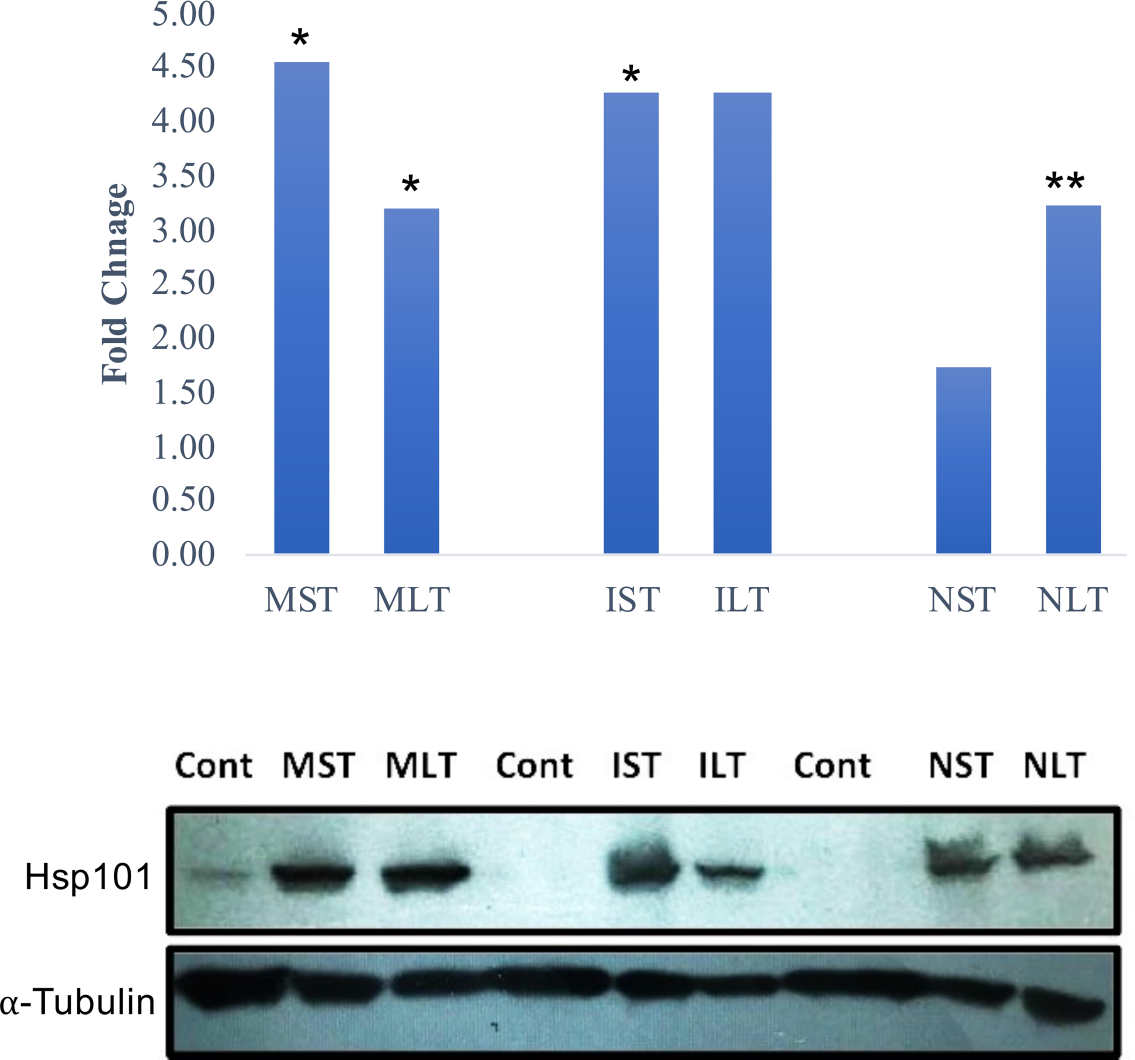
Hsp101 expression profile in Moroberekan, IR64 and N22 rice types post short-term (ST) and long-term (LT) heat stress conditions. The upper panel shows Hsp101 protein expression found in proteome study with significance (*- p value<0.05; **- p value< 0.01). The lower panel shows western blotting for Hsp101 in the anther samples of Moroberekan, IR64 and N22 rice types. Alpha-Tubulin was used as a loading control for the blot to normalize the protein levels detected across the gel. ST, Short term heat stress; LT, Long-term heat stress; MC, Moroberekan control; MST, Moroberekan ST_HS; MLT, Moroberekan LT_HS, IC, IR64 control; IST, IR64 ST_HS; ILT, IR64 LT_HS, NC, N22 control; NST, N22 ST_HS; NLT, N22 LT_HS.

Cytosolic ClpB/HSP100 family proteins are molecular chaperones that promote the renaturation of protein aggregates and are required to develop acquired thermotolerance (Hong and Vierling, 2000). To validate the protein abundance of Hsp 101, a western blot analysis of control and heat-stressed anther tissues was performed. Moroberekan and IR64 showed higher levels of Hsp101 than N22 under ST_HS (**Figure 3**, upper panel). Similarly, the Hsp101 protein showed higher levels in Morobereken and IR64 than N22 (**Figure 3**, lower panel). 

### 3.4 Functional regulation of the differentially expressed proteins (DEPs)

**Figure 4:**
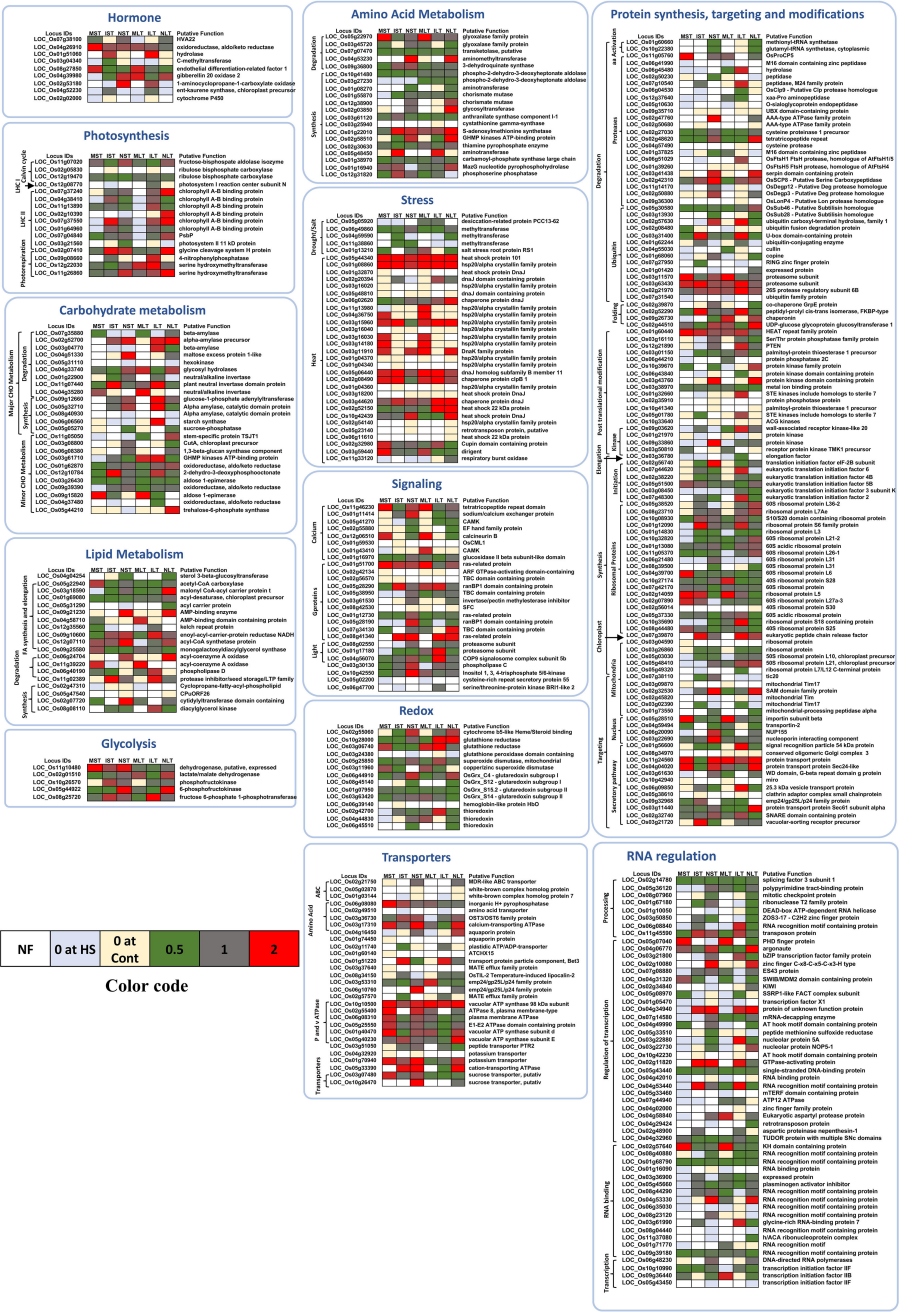
Heat maps depicting the relative abundance of proteins related to different pathways. The proteins significantly changed and differentially abundant in at least one genotype and at least in one condition are shown here. The color bar indicates the color codes used in making the heat map. White color- protein not found (NF); light blue- not found in HS; yellow- exclusively found in HS; green- downregulated (below 0.5 folds); grey- no change; red- upregulated (above 2 folds). ST, Short term heat stress; LT, Long-term heat stress; MC, Moroberekan control; MST, Moroberekan ST_HS; MLT, Moroberekan LT_HS; IC, IR64 control; IST, IR64 ST_HS; ILT, IR64 LT_HS; NC, N22 control; NST, N22 ST_HS; NLT, N22 LT_HS; UP, upregulated; DN, downregulated.

The relative abundance of the selected proteins differentially expressed in at least one of the rice genotypes and at least one condition related to diverse cellular pathways was used for constructing the heat maps for their relative expression (**Figure 4**). In amino acid metabolism, most of the proteins were downregulated. S-adenosylmethionine synthetase was upregulated in all the rice genotypes except in MST. Amino methyltransferase, a glycine cleavage T family protein, was upregulated in both NST and NLT but downregulated in MST, IST and ILT. Phospho-2-dehydro-3-deoxyheptonate aldolase was downregulated in all the rice genotypes. GHMP kinase and ATP binding protein were specifically upregulated in NST and NLT. Several stress-associated genes were upregulated. These included Hsp20/alpha crystallin family protein, DnaK family protein, DnaJ homolog subfamily B member 11 and Hsp101. Genotype-specific Hsp20/alpha-crystallin family protein levels were identified in ST_HS and LT_HS of Moroberekan, IR64 and N22 (**Figure 4**). On the other hand, the proteins in the biotic and drought/salt categories were down-regulated.

HVA22 (ABA and stress-inducible gene) was downregulated in MST, and no change was noted in NST and NLT. Endothelial differentiation-related factor 1 was upregulated in Moroberekan and downregulated in IR64 and N22. Most of the DEPs involved in fatty acid biosynthesis were unchanged or downregulated. Interestingly, most ribosomal proteins were downregulated in the three rice genotypes in all conditions (**Figure 4**). However, a few ribosomal proteins, including 60S ribosomal protein subunits, 60S ribosomal protein L36-2 and ribosomal protein L7Ae were heat induced in NLT. Proteins related to post-translational modification were also heat inducible, especially in the case of NLT. Most photosynthesis-related proteins were downregulated except for a few chlorophyll-binding proteins, specifically in NLT.

### 3.5 Gene ontology (GO) enrichment analysis of the DEPs

**Figure 5:**
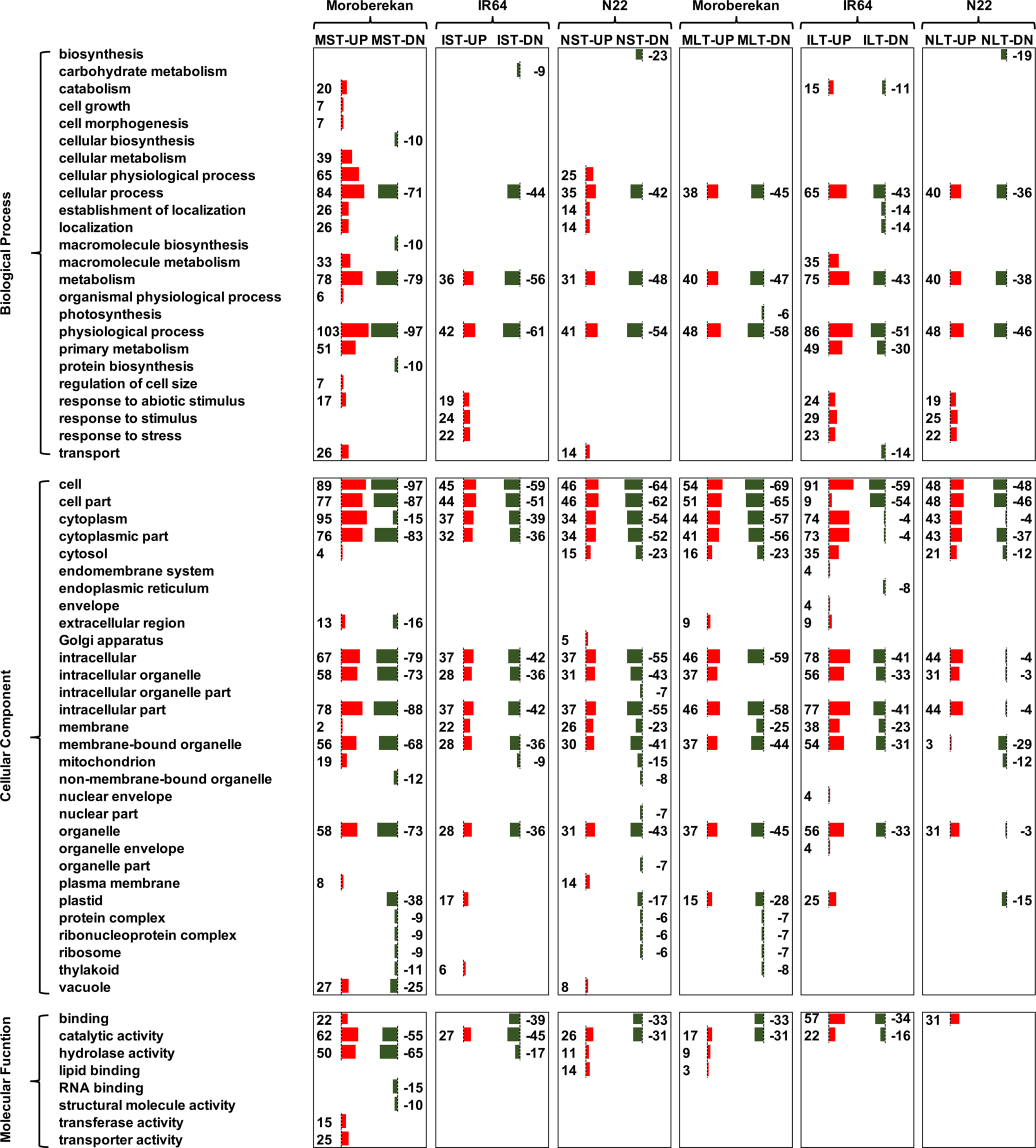
GO term enrichment analysis using RiceNetDB for the proteins up- and down regulated in short-term (ST) and long-term (LT) heat stress conditions in Moroberekan, IR64 and N22 rice genotypes. The numbers are indicative of the proteins enriched for each GO term.

GO enrichment analysis determined that three major biological processes (BP): cellular process, metabolism, and physiological process were enriched (**Figure 5**). These terms were enriched in both up-and down-regulated DEPs of all three rice genotypes, except the cellular process category which was not represented in IST-UP (upregulated in IST of IR64). In response to the abiotic stimulus category, entries were identified in MST-UP and IST-UP, while no entries were found for it in NST-UP. Interestingly, the biological process of transport was enriched in MST-UP (with 26 proteins) and NST-UP (with 14 proteins) and ILT-DN. In the molecular function (MF), the transport category was enriched in MST-UP. In the cellular component, GO terms related to plastids, protein complexes, ribonucleoprotein, ribosomes, and thylakoids were enriched in MST-DN, NST-DN and MLT-DN. GO ontology of BP: protein biosynthesis and macromolecular biosynthesis were enriched in MST-DN only. This trend was reflected in enriched MF: structural molecule activity and RNA binding of MST-DN. The vacuole was enriched in MST-UP and MST-DN and, to a small extent, in NST-UP.

**Figure 6:**
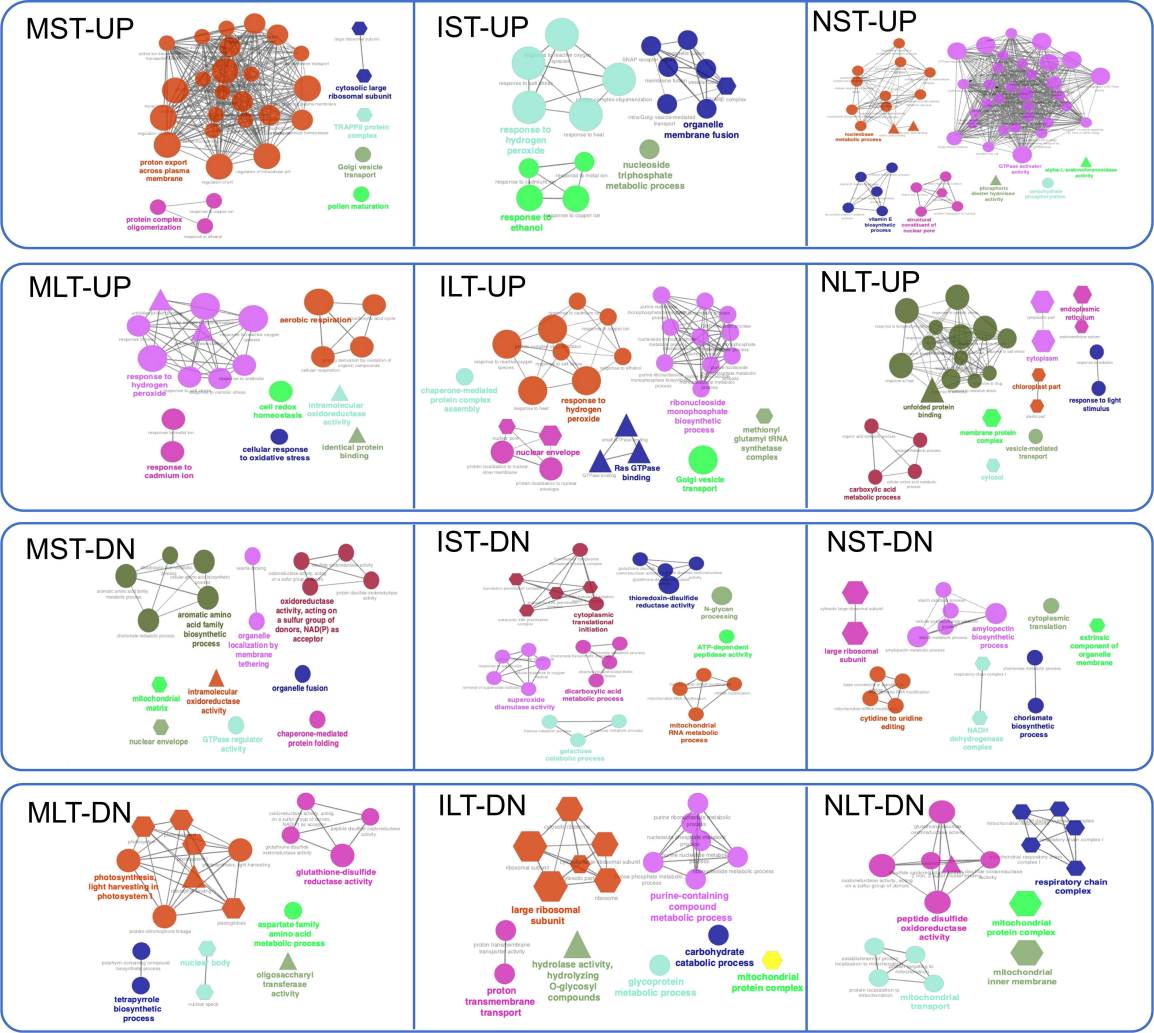
Gene Ontology (GO) network analysis of differentially abundant proteins. GO term enrichment network for the up-and down-regulated proteins in Moroberekan, IR64 and N22 short-term and long-term heat stress conditions was created using Cytoscape software (Version 3.7.1) with ClueGO (Version 2.5.4) plug-in. The colored circles represent the biological function, triangles represent the molecular function, and hexagons represent the cellular components hierarchically. ST, Short term HS; LT, Long, term heat stress; MC, Moroberekan control; MST, Moroberekan ST_HS; MLT, Moroberekan LT_HS; IC, IR64 control; IST, IR64 ST_HS; ILT, IR64 LT_HS; NC, N22 control; NST, N22 ST_HS; NLT, N22 LT_HS; UP, upregulated; DN, downregulated.

The catalytic activity was noted in all treatments except in protein regulation of LT-HS. To further interpret the biological relevance of DEPs, GO terms and pathways were functionally grouped in ClueGO (**Figure 6**). ClueGO integrates GO terms and KEGG pathways, generating a functionally organized GO/pathway network. In MST-UP proteins, a network of proton export across the plasma membrane was identified. A major network in the N22 proteins NST-UP set was GTPase activator activity and vitamin E biosynthetic process. GTPases play an essential role in vesicular trafficking and act as molecular switches regulating plant developmental processes, including embryonic development and reproduction. Proteins in NLT-UP were enriched in unfolded protein binding process and cellular components cytosol, endoplasmic reticulum (ER) and chloroplast. Accumulation of misfolded proteins is at the core of HS in all cellular compartments, and in the ER, it activates a signaling unfolded protein response (UPR) pathway. The upregulation of unfolded protein binding process is in line with the reports showing components of the endoplasmic reticulum-folding machinery and UPR is upregulated at specific stages of pollen development, pollen germination, polar tube growth and fertilization (Fragkostefanakis et al., 2016; Chaturvedi et al., 2021).

### 3.6 Interaction network analysis of the identified proteome

**Figure 7:**
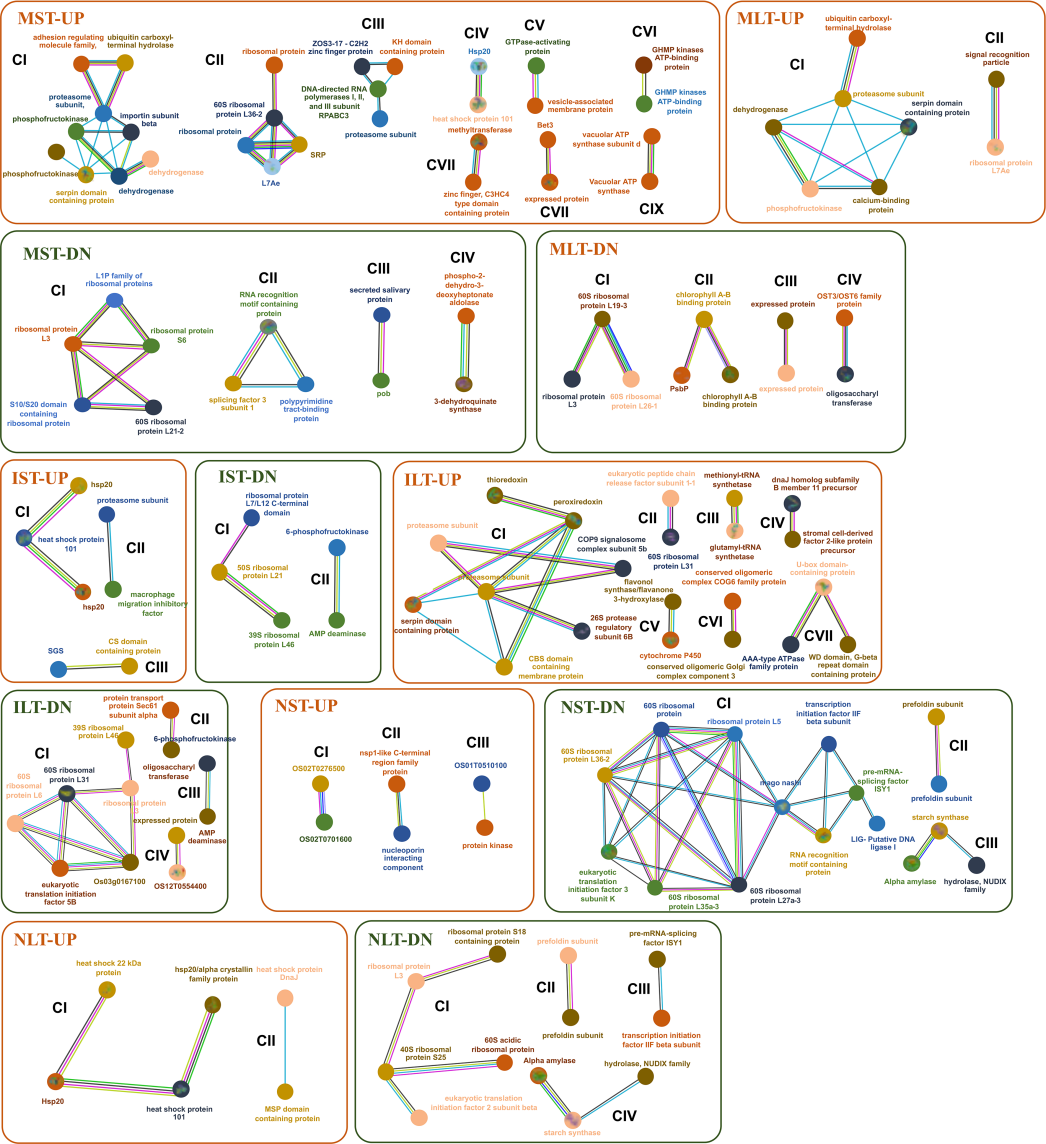
Protein interaction network profiles represent up- and down-regulated protein interactions in Moroberekan, IR64 and N22 in short-term heat stress conditions. The parameter was restricted with the highest confidence to 0.900 neglecting the single molecules found in the interaction map to best predict the interactions. Each circle represents a protein, and lines represent the type of evidence available for finding interactions between the two proteins. Redline- the presence of fusion evidence; Green line- neighborhood evidence; Blue line- cooccurrence evidence; Purple line- experimental evidence; Yellow line- text mining evidence; Light blue line- database evidence; Black line- co-expression evidence. ST, Short term heat stress; LT, Long-term heat stress; MC, Moroberekan control; MST, Moroberekan ST_HS; MLT, Moroberekan LT_HS, IC, IR64 control; IST, IR64 ST_HS; ILT, IR64 LT_HS; NC, N22 control; NST, N22 ST_HS; NLT, N22 LT_HS; UP, upregulated; DN, downregulated.

The STRING database was used to identify the, interactions among proteins in three rice genotypes. Different functional clusters emerged among the interacting proteins. Only selected interactions with more than 0.9 confidence are shown (**Figure 7**). In the MST-UP, nine distinct network clusters were noted (CI-CIX). These clusters included the respiratory metabolism cluster (CI), ribosomal proteins cluster (CII), and Hsps cluster (CIV). In the MST-DN, four distinct clusters were observed, including ribosomal protein (CI) and RNA modification (CII). The IST-UP revealed three clusters of interactions, including an Hsp cluster (CI). IST-DN category showed two clusters, one of which was the ribosomal proteins cluster. In both NST-UP and NST-DN, three clusters were identified. The NST-DN category contained a large cluster of ribosomal proteins, RNA splicing (CI) and starch metabolism (CIII). MLT-UP and MLT-DN two and four clusters were identified, respectively. The MLT-DN contained a cluster of ribosomal proteins (CI). ILT-UP and ILT-DN contained seven and four clusters of protein-protein interactions, respectively. ILT-UP had a proteasome proteins cluster (CI), whereas ILT-DN had a ribosomal protein interaction cluster (CI). In NLT-UP, two clusters were observed, both of which were of Hsps (CI and CII). In NLT-DN, four protein interaction clusters were noted, including one big ribosomal protein cluster (CI), one RNA splicing cluster (CIII) and one starch metabolism cluster (CIV). In the NLT-UP, cluster CI contained chaperone protein ClpB1 (Os05g44340) with 26.7 kDa sHsp (Os03g14180) and 18.0 kDa class II sHsp (Os01g08860), which further interacted with 24.1 kDa mitochondrial sHsp (Os02g52150). Similarly, 24.1 kDa sHsp was found in anther proteome of N22 in an earlier study, and its higher accumulation was linked to better thermotolerance of N22 (Jagadish et al., 2010).

## 4 Discussion

Heat stress response at the reproductive stage of three rice genotypes Moroberekan, IR64, and N22 demonstrate differential effects: Moroberekan is heat-sensitive, IR64 is moderately heat-tolerant, and N22 is heat-tolerant (Jagadish et al., 2010). In the previous study, it has been reported that Nipponbare and N22 rice seedlings demonstrate differential phenotype and protein regulation in response to heat stress under basal (unprimed heat stress regime) as well as short-term and long-term acquired (primed heat stress regimes) thermotolerance assays (Lin et al., 2014). The present study attempts to understand proteome regulations associated with short-term and long-term HS responses in three rice genotypes. Here in N22 higher levels of 6-phosphofructokinase and fructose 6-phospahate 1-phosphotransferase were identified compared to Moroberekan indicating that glycolytic activity was enhanced in N22 under heat-stress conditions. This data were consistent with the previous study were N22 and Moroberekan were compared at metabolome and transcriptome levels (Li et al., 2015). The latter study found the higher expression of Hsp100 in Moroberekan, which agrees with our findings. Jagadish and coworkers also reported that IR64 and N22 were highly induced for putative low molecular weight HSPs (Jagadish et al., 2010). In the current study, we observed a similar response in the case of N22 and IR64 where many of the small sHSPs were induced under heat stress conditions.

Despite having a higher number of proteins in N22, MST showed a higher number of significantly altered proteins (734 proteins) compared to IST (243 proteins) and NST (272 proteins). Several changes in protein levels in different rice genotypes are linked to reproductive processes (**Figure S2**). For example, MADS2 (Os01g66030) regulation in NST-DN, NLT-DN and MST-UP. MADS2 interacts with OsMADS16 and functions in lodicule and stamen development (Kong et al., 2019). The presence of expansins (4 proteins) in the MST-UP suggests their significance in reproduction as the knockdown of Os10g44710 (beta-expansin 2; EXPB2) was reported to cause a sterile phenotype in rice (Huang et al., 2018). Expansins are involved in cell wall loosening and its extension that facilitates the pollen tube penetration through the stigma and style (Tabuchi et al., 2011). MLT-DN was enriched by cellular component-plastid (34 proteins), cytosol (23 proteins), ribosome (8 proteins) and molecular function _binding (24 proteins) and translation (8 proteins) processes. Accordingly, proteins related to these processes, like ribosomal proteins, RNA binding proteins, photosystem and chlorophyll a-b binding proteins, were down-regulated. OsPsbS1 (Os01g64960) in MST-DN and MLT-DN exert control over the CO_2_ assimilation rate in fluctuating light in rice (Hubbart et al., 2012). Aspartic protease (LOC_Os04g58840) and cysteine protease CP1 (Os04g57490) were present in ILT_UP. These proteases are essential for male fertility (Huang et al., 2013). In the mutant plants of aspartic protease pollen maturation was normal, but germination was hampered (Huang et al., 2013). CP1 suppressed mutant showed a significant defect in pollen development. Rice apoptosis inhibitor 5 (LOC_Os02g20930, API5) was downregulated in MST, resulting in delayed degeneration of the tapetum due to inhibition of the tapetal programmed cell death (PCD) process leading to defects in the formation of male gametophyte. Interestingly, OsAPI5 interacts with two DEAD-box ATP-dependent RNA helicases, API5-INTERACTING PROTEIN1 (AIP1) and AIP2 form dimers that interact directly with the promoter region of CP1 gene (Li et al., 2011). In NST-DN, Os03g07140 [male sterility 2; MS2/defective pollen wall (DPW), a fatty acyl-CoA reductase that produces 1-hexadecanol] was identified. This protein is expressed in both tapetal cells and microspores during anther development. DPW participates in a conserved step of primary fatty alcohol synthesis for anther cuticle and pollen sporopollenin biosynthesis in monocots and dicots. Male sterile mutant, *dpw*, displays defective anther development and degenerated pollen grains with an irregular exine (Shi et al., 2011).

### 4.1 Regulation of HSPs in three rice genotypes

In all three rice genotypes, different Hsps were highly induced. Hsps showed genotype - specific protein abundance pattern which can also be used as genotype-specific markers for the heat stress response. The abundance of Hsp20, chaperone protein DnaJ homolog subfamily B member Os05g06440.1 and Hsp101 (Os05g44340) were upregulated across all the three genotypes under stress condition. Hsp101 is an important protein in imparting thermotolerance in bacteria, yeast and Arabidopsis (Kumar et al., 2016; Kumar et al., 2020; Tiwari et al., 2021). The introduction of *Arabidopsis thaliana* Hsp101 (AtHsp101) into rice made the host variety more thermotolerant (Katiyar-Agarwal et al., 2003). Hsp20 provide thermotolerance to various levels of life forms such as bacteria and plants (Sarkar et al., 2009). Hsp20 co-aggregate with misfolded proteins (Sarkar et al., 2009; Kumar et al., 2015; Ungelenk et al., 2016; Sarkar et al., 2020). Furthermore, ATP-dependent Hsp70 and Hsp100 chaperones facilitate the solubilization and refolding of protein aggregates (Cherkasov et al., 2013; McLoughlin et al., 2016; Merret et al., 2017).

In NLT, upregulated proteins demonstrated a significant interaction with Hsps (**Figure 6**). This interaction was not enriched in ILT and MLT. A major interaction with the ribosomal proteins suggests reduction in translation during heat stress. Other major interactions with proteasome and dehydrogenase also indicate that MST is involved in more protein degradation. Heat-induced ribosome inhibits pre-rRNA processing which triggers the imbalance in ribosomal profiles in *Arabidopsis thaliana* (Merret et al., 2015; Darriere et al., 2022).

Another category of stress proteins is related to carbohydrate metabolism. N22 was enriched in proteins such as trehalose-6-phosphate synthase, trehalose synthase, and haloacid dehalogenase-like hydrolase. The trehalose accumulation in rice protects plants from various stresses (Benaroudj et al., 2001). HS affects rice sugar partitioning. It appears that heat-tolerant plant types are better at preserving starch and sugar levels in pollen, which helps them germinate at higher temperatures (Pressman et al., 2002; Firon et al., 2006; Chaturvedi et al., 2015; Jegadeesan et al., 2018). Protein import into the nucleus is another process enriched in N22. Hsfs and other transcription factors are dynamically transported across the nucleus to induce the expression of Hsps and other stress-responsive genes (Scharf et al., 2012). This may indicate more efficient trafficking in N22 under heat stress conditions.

### 4.2 Differential regulation of hormone metabolism and signaling

Thermotolerance involves different signaling pathways where phytohormones play important roles. HS leads to the abscission of reproductive organs due to increased abscisic acid and ethylene levels and reduced levels of auxins (Binder and Patterson, 2009). Also, the alterations in auxin biosynthesis and cytokinin content reduces pollen sterility and kernel filling, respectively, in cereals (Banowetz et al., 1999; Sakata et al., 2010). In the present study, DEPs involved in hormone metabolism were mostly downregulated under HS conditions. HVA22 was specifically induced in IST and ILT but downregulated in MST and MLT, and no change was noted in NST and NLT. This protein acts downstream of GAMyb and activates programmed cell death and other GA-mediated processes (Guo and Ho, 2008). Another protein, CYTOCHROME P450 51G1 (LOC_Os11g32240.1), was highly upregulated in IST, ILT and NLT. This protein participates in brassinosteroid biosynthesis. The application of brassinolide promotes panicle ripening in rice (Saka et al., 2015). Also, brassinolide is known for its induced resistance to abiotic stress in plants.

### 4.3 Regulation of amino acid metabolism in three rice genotypes

Our analysis revealed significant changes in major cellular processes and pathways associated with stress regimes and rice genotypes like amino acid metabolism, maintenance of cellular homeostasis and photosynthesis (**Figure 4**). The amino acid metabolism is a major process affected by HS (Wang et al., 2018). In N22, higher number of proteins involved in amino acid synthesis were upregulated. S-adenosyl methionine (SAM) synthetase protein participates in various biological processes and methionine metabolic pathways. This protein was specifically downregulated in MST. Increased levels of this protein were noted in NST and NLT. This protein also plays a key role in pollen tube growth in the mutant of methionine adenosyltranferase3 (MAT3, one of the four kinds of SAM synthetase genes in *Arabidopsis*) (Chen et al., 2016). Some amino acid metabolism proteins like aminomethyltransferase, chorismate mutase, and cystathionine gamma synthase showed genotype- specific responses but not found in Moroberekan. This change in amino acid metabolism could be linked to the genetic variations in heat tolerance in rice.

### 4.4 Differentially regulated proteins enhancing stress tolerance in the N22 genotype

Proteins upregulated in NLT were majorly enriched in biological processes like unfolded protein binding (**Figure 5**). This process is critical for repairing unfolded or damaged proteins formed during HS (McLoughlin et al., 2019). Furthermore, proteins involved in carboxylic acid metabolism, including those involved in amino acid metabolism, were enriched under NLT-UP. This indicates that N22 anthers can efficiently maintain their energy metabolism under heat stress conditions. The pollen tissues at the mature stage are metabolically active and need energy and new proteins for the successful fertilization (Chaturvedi et al., 2013; Selinski and Scheibe, 2014). Proteins associated with aerobic respiration enrichment were found in MLT-UP, implicating an efficient TCA cycle for energy production. A major process noted in NST-UP was the GTPase activator. GTP metabolism is involved in protein trafficking (Hutagalung and Novick, 2011). This process may be important for maintaining cellular homeostasis during HS. It was also found that NST-UP was enriched in proteins involved in vitamin E biosynthesis (**Figure 5**). Vitamin E protects photosystem II against heat and drought stresses (Havaux et al., 2005).

The amount of information available on photosynthesis-related proteins in rice anthers is limited. Several proteins related to light-harvesting complex II and photorespiration were upregulated in N22, especially in NLT. The chlorophyll a-b binding protein is key to balancing the excitation energy between the two photosystems and was found upregulated in NST. Considering the importance of ABC transporter in pollen exine formation and pollen-pistil interactions (Chang et al., 2016), ABC-type transporters found in both NST and NLT demonstrate relevance in the developmental process.

## 5 Conclusion

**Figure 8:**
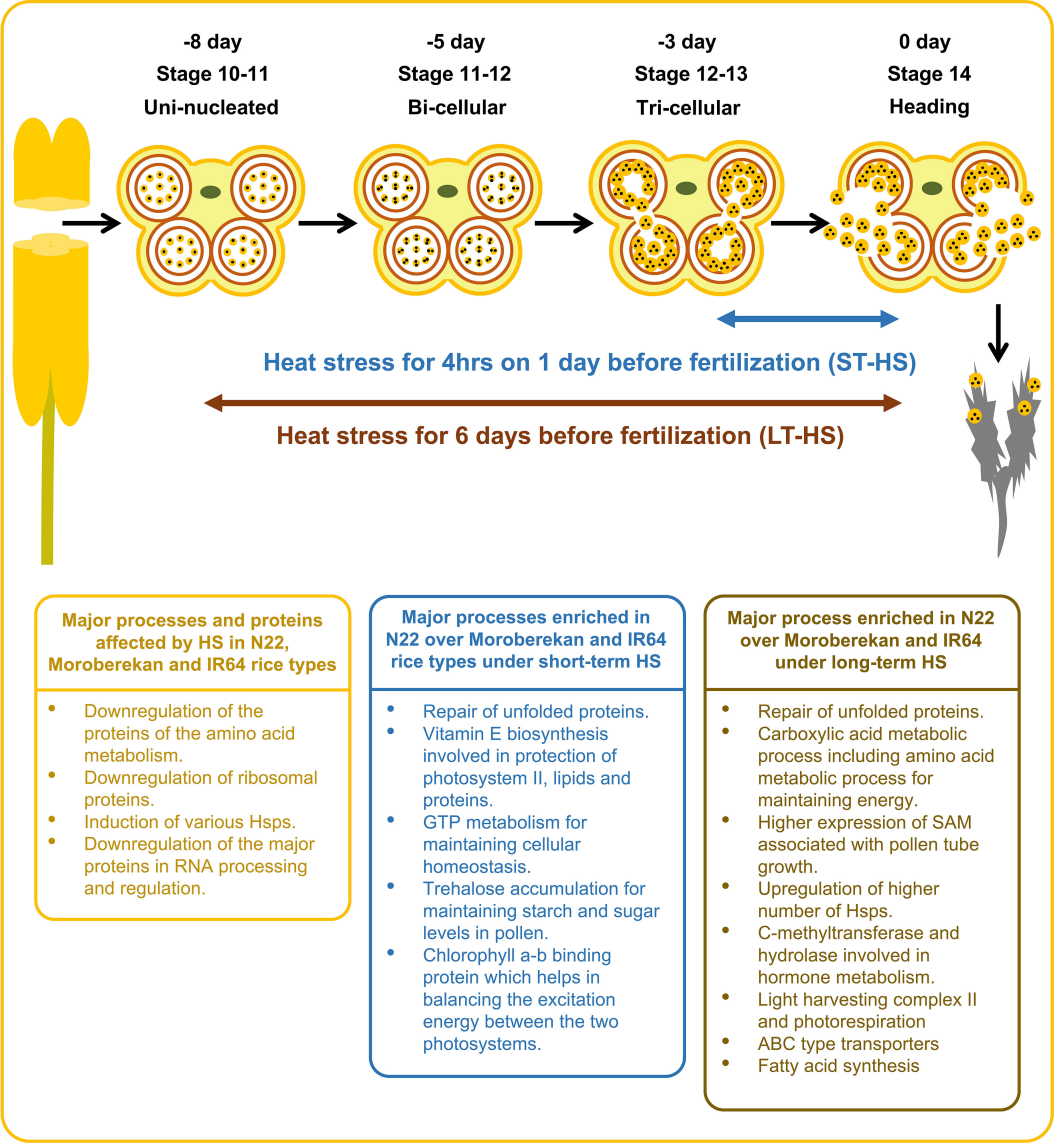
Illustration showing the effects of heat stress on major biological processes and the enriched biological processes in N22 over Moroberekan and IR64 in response to short-term and long-term HS conditions. ST, Short term heat stress and LT, Long-term heat stress conditions.

This study highlights the proteins affected by HS in N22, Moroberekan and IR64 rice genotypes (**Figure 8**). It also demonstrates the biological activities enriched in N22 over Moroberekan and IR64 under short-term and long-term HS. We identified that the major processes critical for N22 tolerance include the repair of unfolded proteins, Vitamin E biosynthesis, and trehalose accumulation processes which help N22 maintain reproduction better than Moroberekan and IR64 under heat stress conditions. We further showed that Hsp20, DnaJ, and Hsp101 are differentially expressed in the three rice genotypes, and these can be critical for N22 thermotolerance at the reproductive stage. Future studies on these proteins may shed light on the genetic mechanisms of heat tolerance in rice. A few quantitative trait loci (QTLs) have been identified in different growth and reproductive stages of rice. A major QTL named Thermotolerance 1 (TT1) encoding α2 subunit of the 26S proteasome and involved in the degradation of ubiquitinated proteins has been found in African rice *Oryza glaberrima* (Yan et al., 2020). Overexpression of a receptor-like kinase (ERECTA) in Arabidopsis, tomato, and rice plants was developed as the potential gene for breeding thermotolerant crops with no growth penalty (Shen et al., 2015). Interestingly, some of the DEPs identified in this analysis may have links to successful reproduction under HS regimes. This study provides a substantial amount of novel information on the proteome regulation in the anthers under heat stress conditions, facilitating a multiomics strategy that could effectively improve crop resilience (Weckwerth et al., 2020).

## Data availability statement

The datasets presented in this study can be found in online repositories. The names of the repository/repositories and accession number(s) can be found in the article/**Supplementary Material**.

## Author contributions

RK and IG performed heat stress treatment, sample generation experiments, and protein extraction to perform western blotting. PC and AGh performed protein extraction, quantification, digestion, LC-MS/MS measurement, peptide and protein identification. RK, NS, PC, and AGr did the data analysis. RK, IG, NS, AGr, AGh, WW, and PC drafted the manuscript. All authors have read and approved the final version of the manuscript.
