# Supplementary figures and images for: Heat-induced proteomic changes in anthers of contrasting rice genotypes under variable stress regimes
Source: Front Plant Sci. 2023 Jan 13;13:1083971. doi: 10.3389/fpls.2022.1083971 (PMC9901367; doi:10.3389/fpls.2022.1083971)

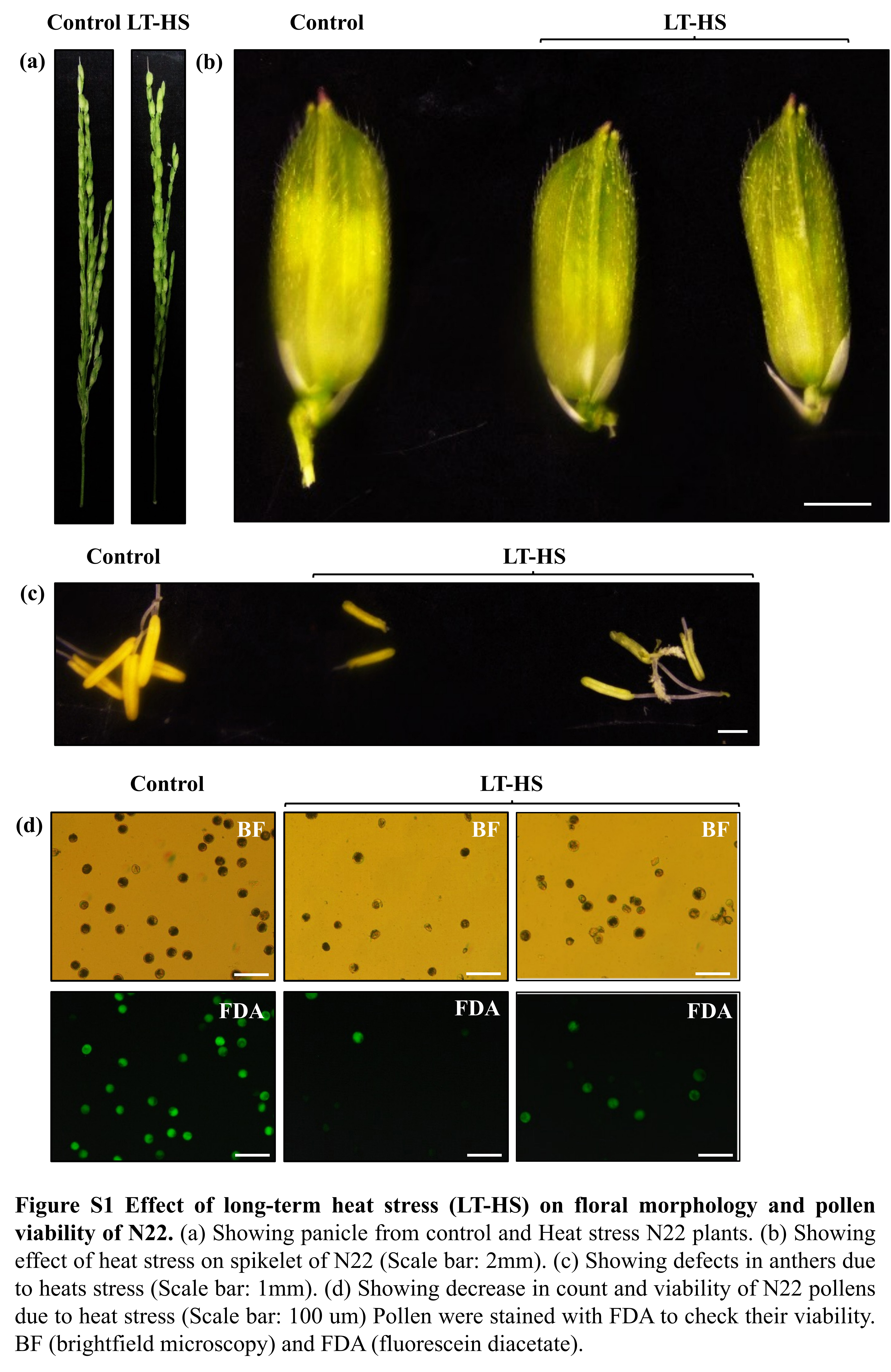

Supplement: Supplementary file 1 [file Image_1.jpeg]

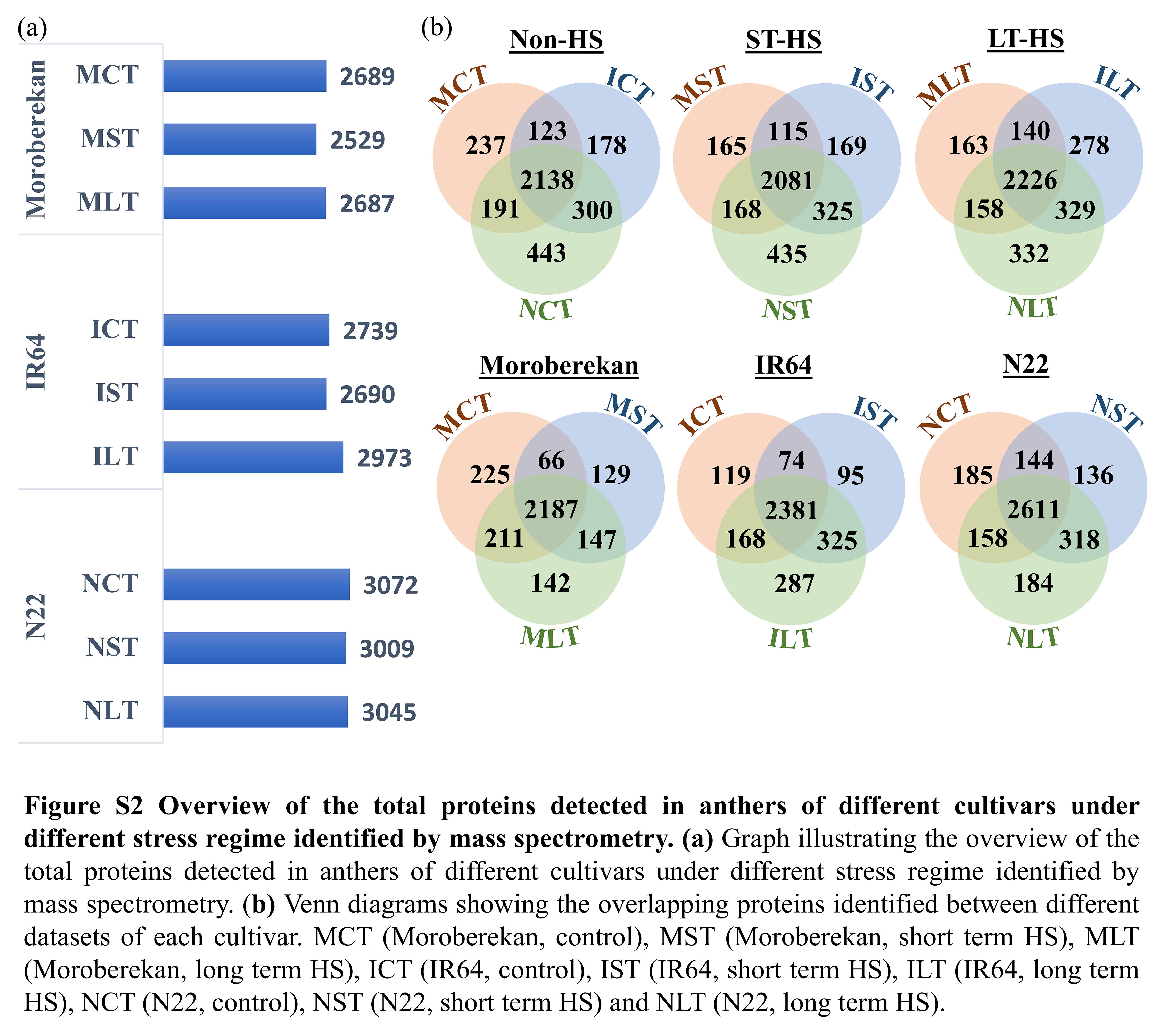

Supplement: Supplementary file 2 [file Image_2.jpeg]

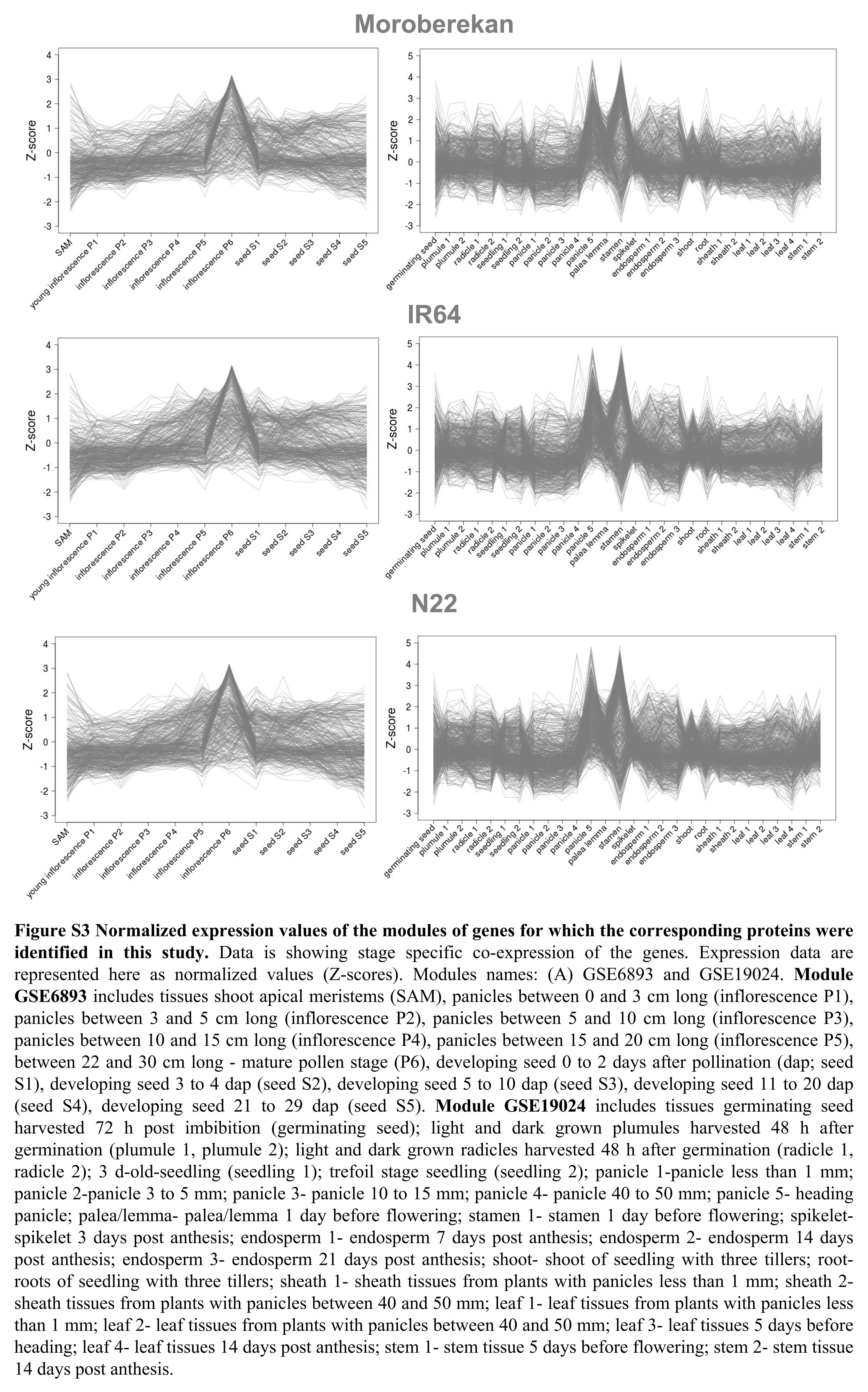

Supplement: Supplementary file 3 [file Image_3.jpeg]

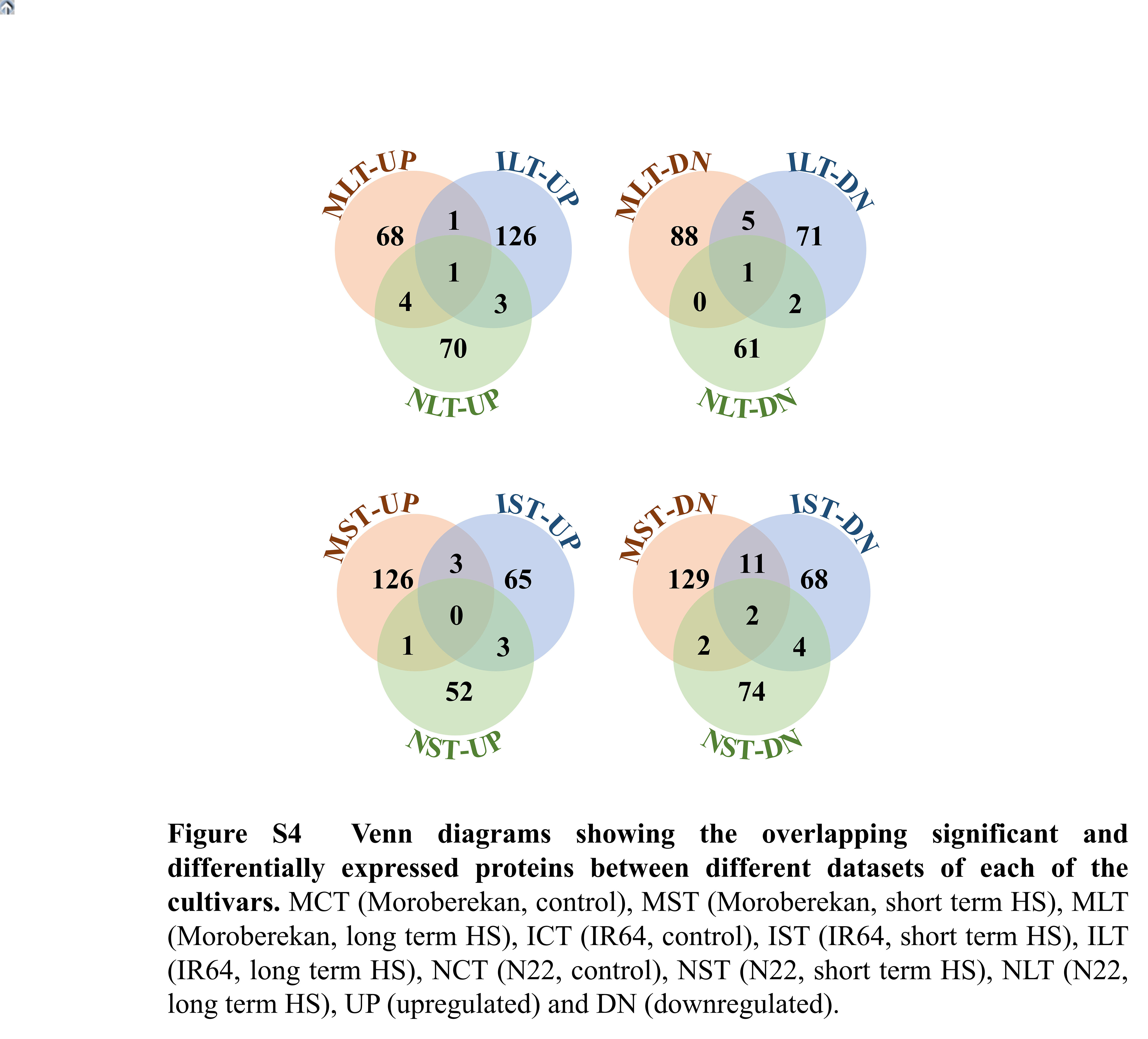

Supplement: Supplementary file 4 [file Image_4.jpeg]
